# Performance Analysis of DCMD Modules Enhanced with 3D-Printed Turbulence Promoters of Various Hydraulic Diameters

**DOI:** 10.3390/membranes15050144

**Published:** 2025-05-10

**Authors:** Chii-Dong Ho, Ming-Shen Chiang, Choon Aun Ng

**Affiliations:** 1 Department of Chemical and Materials Engineering, Tamkang University, Tamsui, New Taipei 251301, Taiwan; 612400498@o365.tku.edu.tw; 2 Faculty of Engineering and Green Technology, Universiti Tunku Abdul Rahman, Kampar 31900, Perak, Malaysia; ngca@utar.edu.my

**Keywords:** DCMD modules, permeate flux, Nusselt number, temperature polarization, 3D-printed turbulence promoters

## Abstract

Theoretical and experimental investigations were conducted to predict permeate flux in direct contact membrane distillation (DCMD) modules equipped with turbulence promoters. These DCMD modules operate at moderate temperatures (45 °C to 60 °C) using a hot saline feed stream while maintaining a constant temperature for the cold inlet stream. The temperature difference between the two streams creates a gradient across the membrane surfaces, leading to thermal energy dissipation due to temperature polarization effects. To address this challenge, 3D-printed turbulence promoters were incorporated into the DCMD modules. Acting as eddy promoters, these structures aim to reduce the temperature polarization effect, thereby enhancing permeate flux and improving pure water productivity. Various designs of promoter-filled channels—with differing array configurations and geometric shapes—were implemented to optimize flow characteristics and further mitigate polarization effects. Theoretical predictions were validated against experimental results across a range of process parameters, including inlet temperatures, volumetric flow rates, hydraulic diameters, and flow configurations, with deviations within 10%. The DCMD module with the inserted 3D-printed turbulence promoters in the flow channel could provide a relative permeate flux enhancement up to 91.73% under the descending diamond-type module in comparison with the module of using the no-promoter-filled channel. The modeling equations demonstrated technical feasibility, particularly with the use of both descending and ascending hydraulic diameters of 3D-printed turbulence promoters inserted into the saline feed stream, as compared to a module using an empty channel.

## 1. Introduction

Membrane distillation (MD) is an advanced water desalination process that leverages a temperature-driven vapor transfer across hydrophobic membranes to separate pure water from saline solutions [1,2]. The driving force for separation is the vapor pressure difference across a microporous hydrophobic membrane, which allows vapor to diffuse through the membrane pores [3,4]. A key advantage of MD is its ability to utilize low-grade heat, enabling operation at relatively low temperatures (40–60 °C), especially when combined with solar heating systems or waste heat from industrial processes. In direct contact membrane distillation (DCMD), the temperature gradient between the hot and cold liquid streams on either side of the membrane generates the vapor pressure difference that drives permeate flux. However, this process is limited by heat transfer resistance [5]. The extent of this limitation is commonly represented by the temperature polarization coefficient (TPC) [6], which quantifies the deviation of the membrane surface temperature from the bulk fluid temperature due to evaporation occurring in the thermal boundary layer of the hot feed stream. This effect reduces the effective temperature gradient across the membrane [7], thereby decreasing the driving force and resulting in lower permeate flux. To analyze and predict the performance of DCMD systems, the dusty gas model (DGM) [8] has been employed. This model captures the complex interactions between simultaneous heat and mass transfer processes across the membrane, including diffusion mechanisms, providing a more accurate assessment of permeate flux in DCMD modules [9].

Both temperature and concentration polarization effects [10] occur within the thermal boundary layer near the membrane surface in direct contact membrane distillation (DCMD) processes, leading to reduced mass transfer performance. The extent of concentration polarization depends on the feed composition and can become increasingly significant under certain conditions [11]. Previous studies have investigated the mass transfer characteristics of vapor transport in membrane distillation modules, aiming to enhance permeate flux efficiency [12]. To mitigate these polarization effects, turbulence promoters have been implemented to suppress the thermal boundary layer and reduce the temperature polarization coefficient (TPC) [13], resulting in improved permeate flux compared to DCMD modules without such enhancements. Various types of turbulence promoters—such as roughened surfaces [14], spacer-filled channels [12], and carbon-fiber spacers [15]—have been widely used in membrane separation processes to generate eddy currents and induce flow disruption, thereby enhancing both heat and mass transfer rates. It is possible to reduce the temperature polarization effect as well as to increase the rate of mass transfer by improving the flow channels [16] in DCMD modules or other commercial-scale pilots and applications, such as a vacuum membrane distillation module [17] and reverse osmosis [18]. However, the integration of turbulence promoters also introduces additional pressure loss in the feed channel [19], which must be compensated for to justify the benefit of increased permeate flux.

The implementation of eddy promoters highlights their hydrodynamic influences on heat and mass transfer mechanisms, demonstrating technical feasibility and considerably improving module performance. The heat-balance modeling, incorporating diffusion and enthalpy flow, was developed theoretically and conducted experimentally on a flat-plate membrane DCMD module in the present study. This suggests that operating the modules with promoter-filled channels generated more turbulence intensity, which reduced temperature polarization resistance and further enhanced permeate fluxes. This resistance is related to the temperature polarization coefficient with the insertion of eddy promoters to interrupt the temperature polarization layers, leading to a higher temperature polarization coefficient value and enhanced permeate flux. The effects of increased turbulence intensity on the permeate flux were assessed using a dimensionless quantity called the heat transfer enhancement factor, and thus, the heat transfer coefficients were analyzed and expressed in terms of the correlated Nusselt number. This enhancement factor correlates inlet hot saline feed temperature and volumetric flow rates under geometric shapes, array configurations, and various hydraulic diameters. Moreover, comparisons on the permeate flux were made between descending and ascending promoter-filled-channel modules with the uniform promoter-filled-channel modules that demonstrated why the descending and ascending promoter-filled-channel designs are preferred. It is believed that the availability of such a simplifying mathematical formulation as developed here for flat-plate DCMD modules is the value in the present work. The same procedure undoubtedly could be used in dealing with many membrane separation processes for further particular applications. In the present study, 3D-printed turbulence promoters [20] were incorporated into parallel-plate DCMD modules, and their influence on permeate flux was examined both theoretically and experimentally. The insertion of these eddy promoters was found to modify hydrodynamic conditions [19], leading to enhanced heat transfer. Additionally, the associated trade-off of increased energy consumption [21] was evaluated to assess both economic viability and technical feasibility. Hydrodynamic investigations under various conditions [22] examined the influence of shear rate variation on disturbing the thermal boundary layer by employing both ascending and descending hydraulic diameters [23], achieving flux improvements with relatively low increases in energy consumption. These 3D-printed promoters not only maintain mechanical strength to prevent membrane vibration but also function effectively as eddy promoters. Two geometric shapes—diamond-type and circle-type—were utilized and arranged in different array configurations, including uniform and varying hydraulic diameters. This work builds upon our previous study involving uniform promoter-filled channels [24], extending the investigation to modules incorporating both ascending and descending promoter-filled channels. The findings demonstrate the technical feasibility and potential advantages of the descending promoter-filled channel design strategy presented in this study.

## 2. Experimental Apparatus and Materials

**Figure 1:**
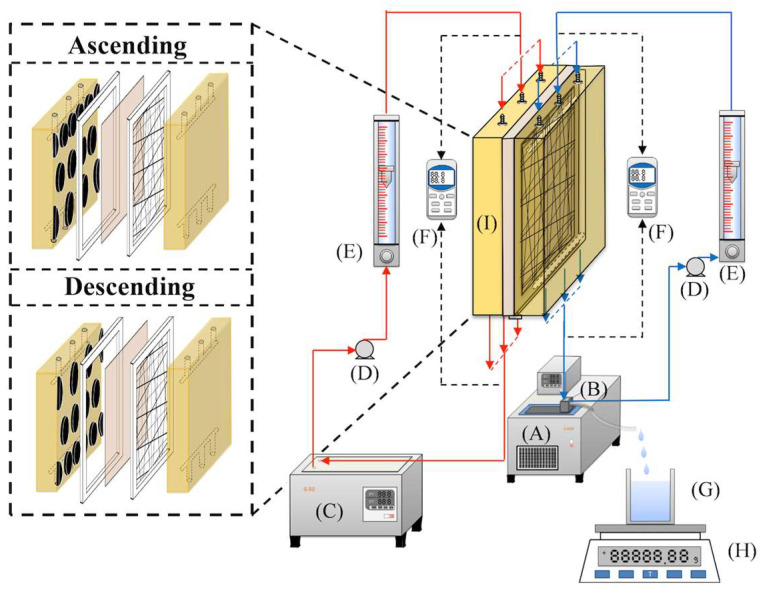
Scheme of a setup used in experiments of flat-plate DCMD modules (A—cold fluid thermostat; B—overflow barrel; C—hot fluid thermostat; D—pump; E—flow meter; F—temperature indicators; G—beaker; H—electric balance; I—the DCMD module).

**Figure 2:**
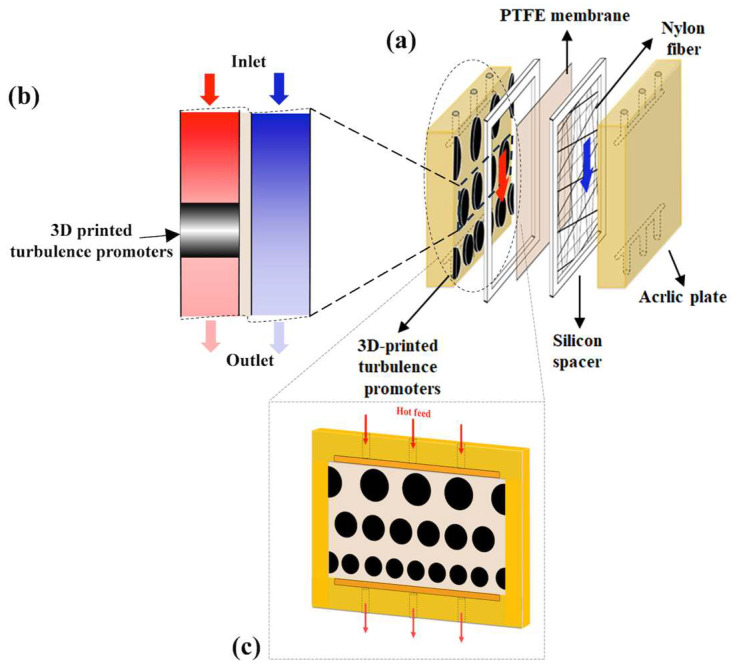
Flat-plate membrane absorption module with inserted 3D-printed turbulence promoters. (**a**) Whole system; (**b**) side-view sector; (**c**) front-view sector.

The schematic configuration and fabrication details of the DCMD modules used for pure water productivity are shown in Figure 1 and Figure 2, representing the flat-plate setup and the promoter-filled module, respectively. The experimental apparatus consists of two parallel-plate channels (L = 0.21 m, *W* = 0.29 m, *d* = 1 mm) separated by a hydrophobic composite membrane made of polytetrafluoroethylene/polypropylene (PTFE/PP, J020A330R, ADVANTEC, Toyo Roshi Kaisha, Ltd., Tokyo, Japan). This membrane has a thermal conductivity of 0.21 W/m K (All-Fluoro Co., Ltd., Taoyuan, Taiwan), and a water vapor permeability resistance was estimated as well [25]. Each channel includes a 1 mm thick silicone rubber gasket affixed to the acrylic plate to form two spacer conduits and ensure leak prevention. The microporous hydrophobic membrane has a nominal pore size of 0.2 µm, porosity of 0.72, and an overall thickness of 130 µm (composed of 98 µm PTFE and 32 µm PP), serving as the medium for vapor flux permeation. The membrane was vertically positioned in the parallel-plate channel to enable two-stream operation: one for the hot saline feed stream incorporating 3D-printed turbulence promoters and the other for the cold stream maintained at 298 K. The integration of 3D-printed geometrically designed turbulence promoters with the DCMD module was proposed to induce eddy currents and enhance hydrodynamic conditions. Two geometric shapes—circular and diamond—were fabricated from polyester elastomer (Polylactic Acid, PLA) using a 3D printer (ATOM 2.5EX, Hsinchu County, Taiwan). Each promoter, acting as eddy promoters, had a thickness of 1 mm and was bonded onto the acrylic plate in contact with the membrane surface in the hot saline feed side using Cyanoacrylate Adhesive (Chang Chun Plastics Co., Ltd., Hsinchu, Taiwan). The cold stream feed channel was supported with a 0.1 mm nylon fiber mesh to prevent membrane vibration and wrinkling. The thicknesses of the channel with/without the implementation of eddy promoters are the same dimension of 1 mm.

An artificial hot saline solution containing 3.5 wt% NaCl was prepared by dissolving inorganic salt (NaCl) in distilled water. Two thermostats were employed to maintain the inlet temperatures of both the hot saline water and the cold pure water streams at their specified values. The saline solution was circulated using a pump (51K40RA-A, ASTK, New Taipei, Taiwan), and its temperature was regulated by thermostats (G-50 and D650, DENG YNG, New Taipei, Taiwan) to maintain preset inlet temperatures of 45 °C, 50 °C, 55 °C, and 60 °C. Inlet and outlet temperatures were monitored using thermometer probes (TM-946, Lutron, New Taipei, Taiwan) installed on both sides of the flat-plate membrane modules. The same flow rates of both the hot saline and cold feed streams were conducted—ranging from 0.1 to 0.4 L/min (0.1, 0.2, 0.3, and 0.4 L/min) during the experimental runs—and were controlled using flow meters (FE-091312-D, Fong-Jei, Hsinchu, Taiwan) and a flow controller (N12031501PC-540, Protec, Brooks Instrument, Hatfield, PA, USA). The cold feed stream with inlet temperature was maintained at 25 °C using a separate temperature controller (FN-0423112-F, Fong-Jei, Hsinchu, Taiwan) until the system reached steady-state conditions. The conductivity of the collected permeate was measured to confirm water purity, with values consistently below 1.5 µs/cm. Comparative analyses of permeate flux were conducted under various operating conditions to evaluate the performance of two DCMD modules—with and without the implementation of eddy promoters.

**Figure 3:**
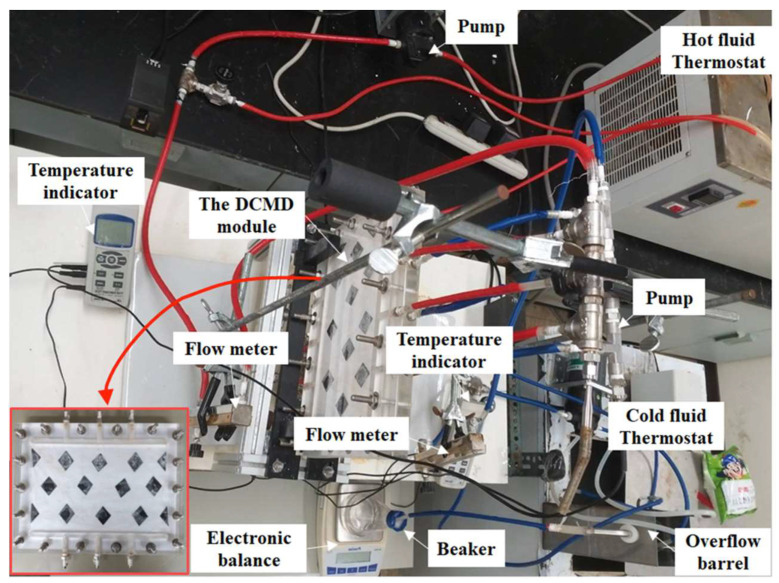
Photographic images of the DCMD experimental apparatus with inserted 3D-printed turbulence promoters (top view).

A photograph of the operating experimental setup for the flat-plate DCMD system is shown in Figure 3. The system features acrylic plates serving as the outer walls of the parallel-plate channel. Comparative experiments were conducted to evaluate permeate flux under various operating conditions using flat-plate membrane distillation modules with and without the incorporation of eddy promoters. The hot saline water exited the membrane module while permeate vapor condensed and collected in the cold stream until the steady-state operation was reached. The collected permeate was measured using an electronic balance (XS 4250C, Precisa Gravimetrics AG, Dietikon, Switzerland), and the data were recorded via a PC. The repeatability of pure water conductivity measurements was always within 5%, enabling reliable determination of vapor permeate flux.

**Figure 4:**
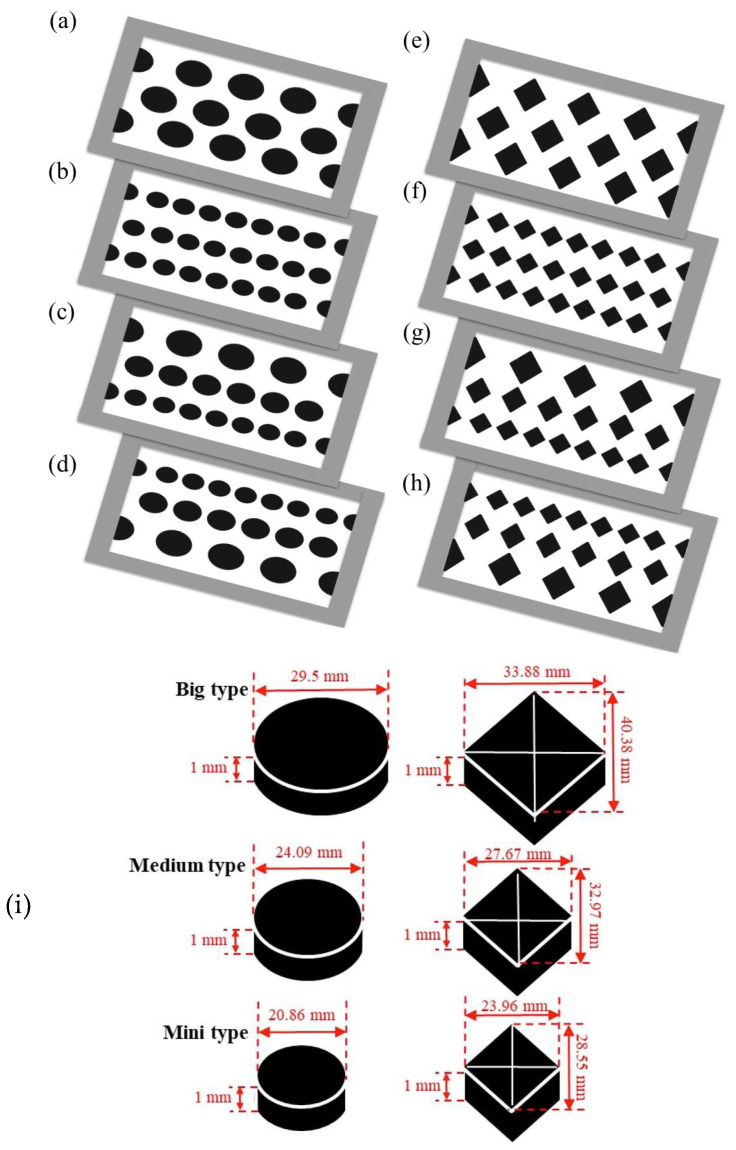
Array configurations of DCMD module with the insertion of various 3D-printed turbulence promoters. (**a**) Big circle; (**b**) mini circle; (**c**) ascending circle sizes; (**d**) descending circle sizes; (**e**) big diamond; (**f**) mini diamond; (**g**) ascending diamond sizes; (**h**) descending diamond sizes; (**i**) the dimensions of the promoters.

Figure 4 presents top-view images of the two geometric shapes—circular-type and diamond-type promoters—arranged on the membrane surface in both uniform and varying hydraulic diameter configurations, including ascending and descending patterns, as shown in Figure 4c,d,g,h, with the dimensions of the promoters in Figure 4i. These promoters partially obstructed the membrane’s transport passages, reducing the effective permeate area by approximately 13%. The implementation of eddy promoters significantly enhanced turbulence intensity within the saline feed channel by disrupting the thermal boundary layer. This effect was particularly pronounced in modules utilizing ascending and descending hydraulic diameter configurations, which contributed to improved heat transfer rate and increased permeate flux. All array configurations were conducted under the co-current mode in the present study.

## 3. Theory and Analysis

### 3.1. Mass Transfer

**Figure 5:**
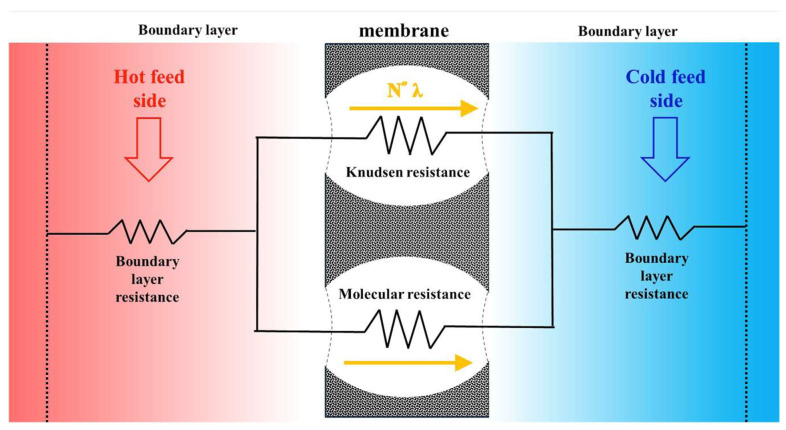
Heat transfer resistances governed by vapor diffusion and enthalpy flow conservation in a flat-plate membrane distillation module.

The performance of the DCMD device depends on the complex interplay between simultaneous heat and mass transfer mechanisms. In this study, a mass-balance model for water vapor transport in the hot saline feed channel of a flat-plate DCMD module—employing a polytetrafluoroethylene/polypropylene (PTFE/PP) membrane—was developed and validated through both theoretical and experimental approaches. This work extends previous research involving uniform promoter widths [24] by incorporating ascending and descending hydraulic diameter configurations in the hot saline feed stream to achieve enhanced permeate flux. Figure 5 illustrates the heat transfer resistances governed by vapor diffusion and enthalpy flow conservation within the DCMD module. The coupled mass transfer behavior was examined to describe the temperature gradient between the hot saline feed and the cold stream. To estimate the permeate flux, an expression for the membrane permeation coefficient ($c_{m}$) was used, which combines the effects of Knudsen and molecular diffusion, as appropriate for microporous hydrophobic membranes [26]. Given the relatively small pore size, the influence of the Poiseuille flow can be neglected [27] and the tortuosity can be estimated using the porosity of the membrane [28]. The product of the membrane permeation coefficient ($c_{m}$) and the transmembrane vapor pressure difference (ΔP) was used to model the permeate flux in the membrane distillation process [4,29].

(1) $$N^{\prime \prime }=c_{m}\Delta P=c_{m}\left[P_{1}^{sat}(T_{1})-P_{2}^{sat}(T_{2})\right]\;{=c}_{m}{\left.\frac{dP}{dT}\right|}_{T_{m}}\left(T_{1}-T_{2}\right)=c_{m}\frac{P_{m}\lambda M_{w}}{RT_{m}^{2}}\left(T_{1}-T_{2}\right)\;$$

and

(2) $$c_{m}={\left(\frac{1}{c_{K}}+\frac{1}{c_{M}}\right)}^{-1}={\left\{{\left[\frac{2\sqrt{8\pi }}{3}\frac{\varepsilon \;r}{\tau \delta_{m}}{\left(\frac{M_{w}}{RT_{m}}\right)}^{1/2}\right]}^{-1}+{\left[{\left|Y_{m}\right|}_{ln}\frac{D_{m}\varepsilon }{\delta_{m}\tau }\frac{M_{w}}{RT_{m}}\right]}^{-1}\right\}}^{-1}\;in\;which\;{\left|Y_{m}\right|}_{ln}=\frac{\left(P_{T}-P_{2}\right)-\left(P_{T}-P_{1}\right)}{P_{T}ln\left[\frac{\left(\left(P_{T}-P_{2}\right)\right)}{\left(\left(P_{T}-P_{1}\right)\right)}\right]},\;\tau =\frac{1}{\varepsilon },\;\;D_{m}=\frac{1\times 10^{-7}T_{m}^{1.75}}{\frac{P_{T}}{101325}M_{w-air}^{0.5}{\left(\sigma_{w}^{\frac{1}{3}}+\sigma_{air}^{\frac{1}{3}}\right)}^{2}},\;M_{w-air}={\left[\left(\frac{1}{M_{w}}\right)+\left(\frac{1}{M_{air}}\right)\right]}^{-1}$$

### 3.2. Heat Transfer

**Figure 6:**
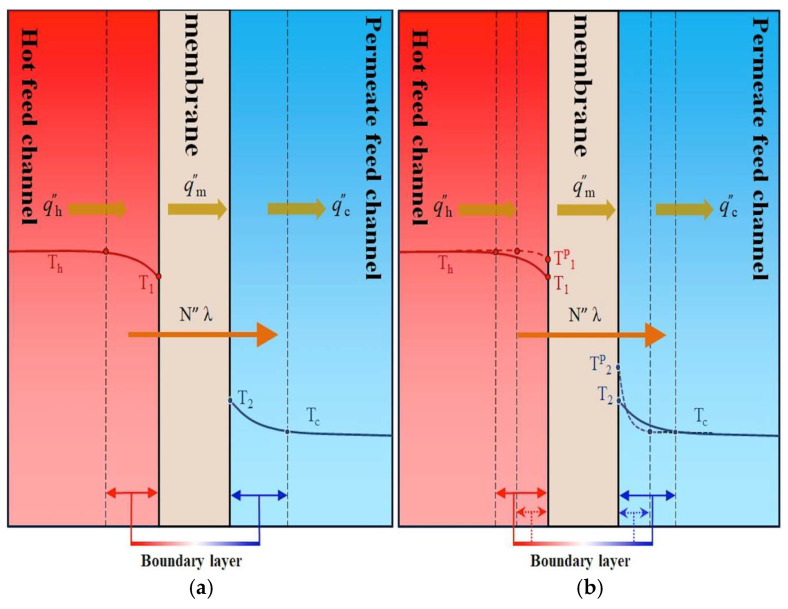
Reduction of thermal boundary polarization layers in the DCMD module. (**a**) Empty channel; (**b**) channel with inserted 3D-printed turbulence promoters.

In the membrane distillation process, water vapor diffuses exclusively through the porous hydrophobic membrane. This process is graphically represented by the bulk temperatures of the feed streams—denoted as $T_{h}$ (hot) and $T_{c}$ (cold)—and the corresponding membrane surface temperatures $T_{1}$ and $T_{2}$, respectively. Regarding Figure 6, one obtains

(3) $$q_{h}^{\prime \prime }=h_{h}(T_{h}-T_{1}),\mathrm{for}\mathrm{the}\mathrm{hot}\mathrm{saline}\mathrm{feed}\mathrm{region}$$

(4) $$q_{c}^{\prime \prime }=h_{c}(T_{2}-T_{c}),\mathrm{for}\mathrm{the}\mathrm{cooling}\mathrm{feed}\mathrm{region}$$

The heat transfer in terms of the overall heat transfer coefficient of the membrane $H_{m}$ including the latent heat across the membrane is defined as

(5) $$\begin{array}{l} q_{m}^{\prime \prime }=N”\lambda +\frac{k_{m}}{\delta_{m}}\;(T_{1}-T_{2})=H_{m}(T_{1}-T_{2})\\ in\;which\;\\ H_{m}=\left\{c_{m}\frac{\left[\left(1-x_{\mathrm{NaCl}}\right)a_{w}P_{2}+P_{1}\right]\lambda^{2}M_{w}}{2RT_{m}^{2}}+\frac{k_{m}}{\delta_{m}}\right\}=\left\{c_{m}\frac{\left[\left(1-x_{\mathrm{NaCl}}\right)\left(1-0.5x_{\mathrm{NaCl}}-10x_{\mathrm{NaCl}}^{2}\right)P_{2}+P_{1}\right]\lambda^{2}M_{w}}{2RT_{m}^{2}}+\frac{k_{m}}{\delta_{m}}\right\} \end{array}$$

where N”λ is categorized as the latent heat of vaporization and $a_{w}$ [30] is the water activity coefficient.

The heat transfers across the hot saline feed stream, membrane, and cold feed stream should be balanced using Equations (3) and (5) ($q_{h}^{\prime \prime }=q_{m}^{\prime \prime }$) and Equations (4) and (5) ($q_{m}^{\prime \prime }=q_{c}^{\prime \prime }$) as follows:

(6) $$h_{h}\left(T_{h}-T_{1}\;\right)=H_{m}\left(T_{1}-T_{2}\right)=h_{c}(T_{2}-T_{c}\;)$$

Solving Equation (6) with respect to *T*_1_, *T*_2_, one obtains

(7) $$T_{1}=\frac{h_{h}T_{h}(H_{m}+h_{c})+{H_{m}h_{c}T}_{c}}{h_{h}H_{m}+h_{c}\left({H_{m}+h}_{h}\right)}$$

(8) $$T_{2}=\frac{h_{c}T_{c}\left({H_{m}+h}_{h}\right)+H_{m}h_{h}T_{h}}{h_{h}H_{m}+h_{c}\left({H_{m}+h}_{h}\right)}$$

The influence of the temperature polarization effect [26] on the device performance of the DCMD module is normally used to indicate the extent of the thermal boundary layer resistance between both hot saline and cooling feed streams, which controls the permeate flux through the membrane; the $\tau_{temp\;}$ is commonly defined as

(9) $$\tau_{temp}=(T_{1}-T_{2})/(T_{h}-T_{c})$$

The insertion of 3D-printed turbulence promoters into the hot saline water compartment increases from the heat transfer coefficient $h_{h}$ (in the module without promoters) to the improved heat transfer coefficient $h_{h}^{p}$ (in the module with promoters). Based on Equations (3)–(5), the schematic diagram in Figure 6a illustrates all heat transfer regions at the microscopic level under steady-state operation. The reduction in the temperature polarization effect resulting from the promoter insertion is demonstrated in Figure 6b. This modification leads to an increased temperature gradient across the membrane surfaces—specifically, ${∆T^{p}(T}_{1}^{p}-T_{2}^{p})>{∆T(T}_{1}-T_{2}$)—thereby enhancing the driving force for heat transfer and increasing vapor flux. The disruption of the laminar thermal boundary layer, along with the generation of intensified vortices or secondary flow patterns, plays a critical role in mitigating the temperature polarization effect by altering the hydrodynamic conditions near the membrane surface.

Substituting both membrane surface temperatures ($T_{1}$ and $T_{2}$) in Equations (7) and (8) into Equation (9), one obtains

(10) $$\tau_{temp}=\frac{h_{h}h_{c}}{h_{h}h_{c}+h_{h}H_{m}+h_{c}H_{m}}$$

The permeate flux was estimated and calculated from Equation (1) and validated by the experimental results by iterating $T_{1}$ and $T_{2}$ and incorporating an initial guess of $h_{h}$ from Equations (7) and (8) until reaching the convergence tolerance.

### 3.3. Theoretical Analyses of the Heat Transfer and Mass Transfer

**Figure 7:**
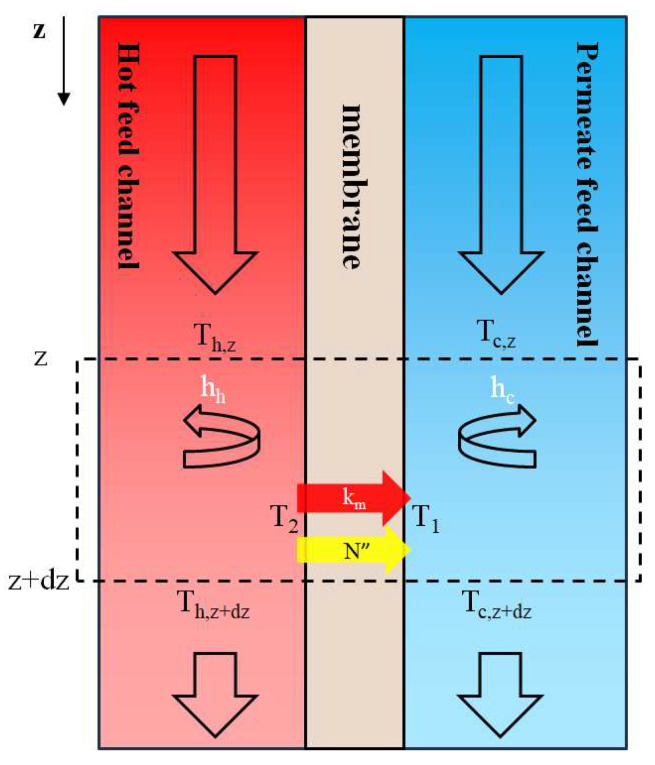
The energy balance made by using the plug–flow description within a finite fluid element in a flat-plate membrane module.

The energy conservation in one-dimensional governing equations was derived according to the plug–flow description, as shown in Figure 7, for solving the temperature distributions of both hot saline and cold feed streams in terms of the temperature polarization coefficient $\tau_{temp}$ as

(11) $$\frac{dT_{h}}{dz}=\frac{-q^{\prime \prime }W}{Q_{h}\;\rho_{h}C_{p,h}}=\frac{-W}{Q_{h}\;\rho_{h}C_{p,h}}H_{m}\tau_{temp}(T_{h}-T_{c})$$

(12) $$\frac{dT_{c}}{dz}=\frac{q^{\prime \prime }W}{Q_{c}\;\rho_{c}C_{p,c}}=\frac{W}{Q_{c}\;\rho_{c}C_{p,c}}H_{m}\tau_{temp}(T_{h}-T_{c})$$

**Figure 8:**
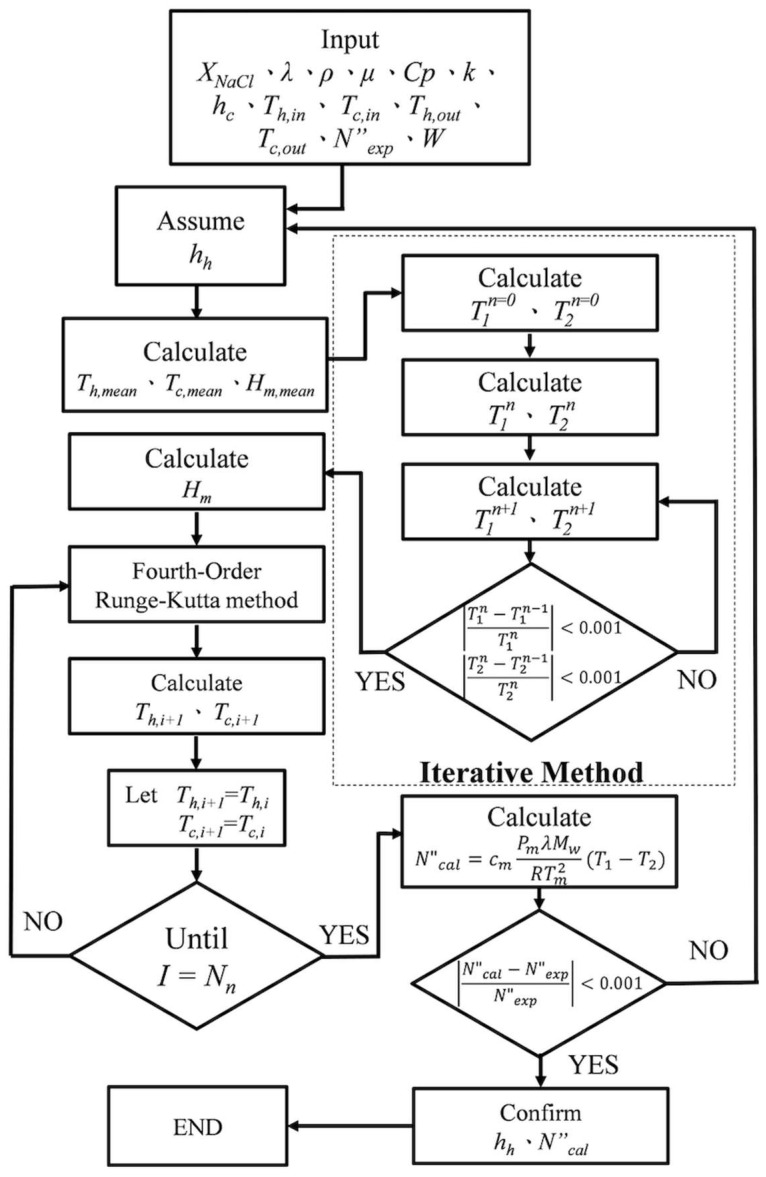
Flow chart for determining temperature distributions in both hot and cold feed streams.

Both $H_{m}$ and $\tau_{temp}$ depend on the coordinate z; two simultaneous ordinary differential equations of Equations (11) and (12) were solved numerically by marching the fourth-order Runge–Kutta method along the length of the module. The equations were iteratively calculated using estimated heat transfer coefficients along the membrane distillation module following the flow chart for determining temperature distributions ($T_{h}$ and $T_{c}$) in both hot and cold feed streams in Figure 8. This numerical approach was used to compute the temperature distributions of both hot and cold feed streams, from which the enhanced permeate flux and corresponding improvements were subsequently determined. Figure 8 shows the calculation of $H_{m}$ during the iteration loop, and Equation (1) was used for calculating $N_{cal.}^{”}$.

The calculation procedure of the heat transfer coefficient is described as follows. First, with the given operation conditions, the heat transfer coefficient is determined from Equations (7) and (8). Next, with the known inlet and outlet temperatures of both hot and cold feed streams, an initial guess of $T_{1}$ (or $T_{2}$) is estimated from Equation (7) once $T_{2}$ (or $T_{1}$) was assumed in Equation (8). With this estimated value of the convective heat transfer coefficient, new values of $T_{1}$ and $T_{2}$ are then repeated from Equations (7) and (8). If the calculated values of $T_{1}$ and $T_{2}$ are not converged, continuous iterating calculation is needed until the last assumed values of membrane surface temperatures meet the finally calculated values. Further, the convective heat transfer coefficient is calculated from Equation (1) to evaluate permeate flux and validated by the experimental results, which are delivered to predict theoretically values not only on the membrane surfaces ($T_{1}$ and $T_{2}$) but also in the hot/cold bulk flows ($T_{h}$ and $T_{c}$) of both hot and cold feed streams, respectively.

The eddy promoters play a crucial role in enhancing and explaining both heat and mass transfer behaviors in DCMD modules. Their implementation enhances turbulence intensity, effectively disturbing the thermal boundary layer and overcoming the temperature polarization effect, which significantly contributes to improved flow characteristics and heat transfer near the membrane surface. The augmented convective heat transfer coefficients were evaluated by comparing them to those in modules using empty channels (without promoters) and across various array configurations of promoter insertion.

The heat transfer enhancement factor, $\alpha^{p}$ [31], is defined as the ratio of the heat transfer rate improvement in a module with embedded turbulence promoters compared to that in a module with no-promoter-filled channels under various array configurations. To calculate the enhancement of the heat transfer rate by implementing a promoter-filled channel in DCMD modules, comparisons were made between modules with inserting turbulence promoters and those without turbulence promoters. Improved heat transfer coefficients were related to the heat transfer enhancement factor and expressed in terms of the correlated Nusselt number. The correlated Nusselt number was integrated into the heat transfer enhancement factor, $\alpha^{p}$, to increase the heat transfer coefficient and reduce the temperature polarization effect, thereby increasing the temperature driving force across the membrane. A simplified relationship [32] between ${Nu}^{p}$ (the Nusselt number for the promoter-filled channel) and ${Nu}_{lam}$ (the Nusselt number for laminar flow in the no-promoter-filled channel) is given by

(13) $$Nu^{p}=\frac{h_{h}^{p}{De}_{h,h}^{h}}{k}=\alpha^{p}Nu_{lam}$$

where $h_{h}^{p}$ is defined as the improved heat transfer coefficient of the hot feed channel with promoter insertion, while the heat transfer equivalent diameter ${De}_{h,h}^{h}\;$ is defined [33] as follows:

(14) $${De}_{h,h}^{h}=\frac{4\left(dW-N_{p}W_{p}D_{p}\right)}{W-N_{p}W_{p}}$$

**Figure 9:**
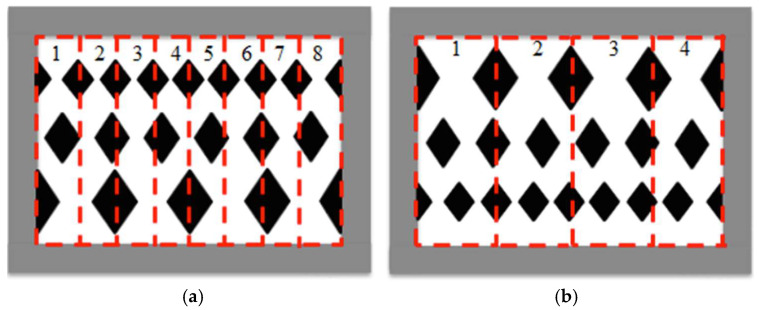
The sketch segment of $N_{P}$ for both ascending and descending diamond-type promoters. (**a**) Ascending array ($N_{P}=8)$; (**b**) descending array ($N_{P}=4)$.

The corelated Nusselt number ${Nu}^{p}$ defines a module with a configuration filled by various promoters and incorporating dimensionless groups into Buckingham’s π theorem while, the Nusselt number ${Nu}_{lam}$ is the membrane distillation module that uses a no-promoter-filled channel under laminar flow operations with a regressed correlation equation, as follows:

(15) $$\alpha^{P}=\frac{{Nu}^{p}}{{Nu}_{lam}}=k_{1}{\left(\frac{W_{p}}{L}\right)}^{k_{2}}{{\left(R_{w}\right)}^{k_{3}}(Re)}^{k_{4}}{\left(Pr\right)}^{k_{5}}$$

where $W_{P}$ and $N_{P}$ represent the pseudo-average width occupied by turbulence promoters in each flow segment and the number of flow segments under various configurations, respectively. $R_{w}$ denotes the ratio of the largest width of each turbulence promoter to the average big-circle promoter width. These parameters are summarized in Table 1 for different promoter-array configurations, and a schematic representation of the segment count $N_{p}$ is shown in Figure 9. This, in turn, indicates a reduction in the temperature polarization effect and an increase in permeate flux across the membrane.

### 3.4. Power Consumption Increment

The hydraulic equivalent diameters $D_{h,h}$ and $D_{h,c}$ for the modules—with embedded 3D-printed turbulence promoters and empty channels on the hot and cold feed sides, respectively—were calculated using the expression 4A/P, where A is the wetted area and P is the wetted perimeter. The average widths of the diamond-type and circle-type turbulence promoters were determined by averaging measurements from 300 and 150 individual promoters, respectively. The hydraulic equivalent diameter $D_{h,h}=\frac{4(dW-{N_{p}W_{p}D}_{p})}{2(d+W+N_{p}D_{p})}$ and $D_{h,c}=\frac{4dW}{2(d+W)}$ of modules with embedding 3D-printed turbulence promoters and no-promoter-filled channel of both hot and cold feed streams, respectively. An unavoidable hydraulic consumption increment is expected and extra energy consumption required due to inserting 3D-printed turbulence promoters into the flow channel, which may be determined using Fanning friction factor ($f_{F}$) [34] while considering only the friction losses to walls of both hot and cold feed streams as follows:

(16) $$H=H_{h}+H_{c}=Q_{h}\rho_{h}lw_{f,h}+Q_{c}\rho_{c}lw_{f,c}$$

(17) $${lw}_{f,h}=\frac{2f_{F,h}{\bar{v}}_{h}^{2}L}{D_{h,h}}$$

The Fanning friction factor can be estimated using a correlation based on the aspect ratio of the channel (α=d/W) [35]:

(18) $$f_{F,h}=\frac{C}{{Re}_{h}},f_{F,c}=\frac{C}{{Re}_{c}}$$

(19) $$C=24\left(1-1.3553\alpha +1.9467\alpha^{2}-1.7012\alpha^{3}+0.9564\alpha^{4}-0.2537\alpha^{5}\right)$$

The percentage of the relative extents $I_{P}$ of the energy consumption increment for the module with inserted eddy promoters is illustrated in comparisons to the module of using an empty channel as

(20) $$I_{P}=\frac{H_{promoter}-H_{empty}}{H_{empty}}\times 100\%$$

where the subscripts of *promoter* and *empty* represent the modules using a promoter-filled channel and an empty channel, respectively.

## 4. Results and Discussions

### 4.1. Diminishing Temperature Polarization Effect by Inserting 3D-Printed Turbulence Promoters

**Figure 10:**
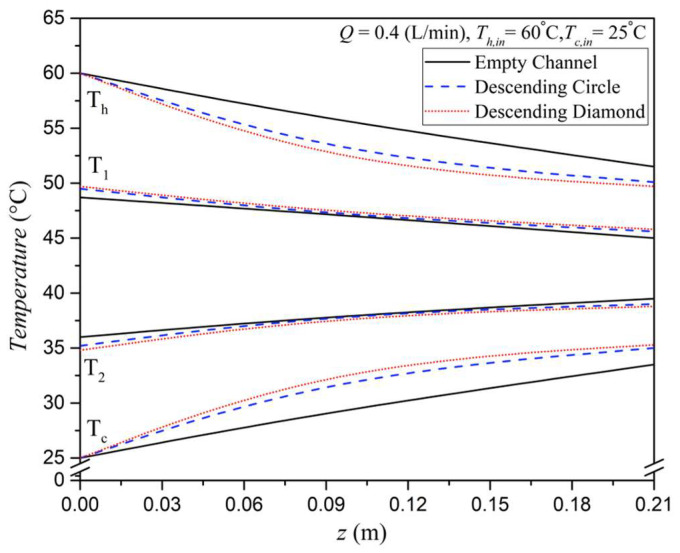
Effects of array configurations on temperature distributions along the module.

Temperature profiles for both the hot saline and cold feed streams were determined by solving two simultaneous ordinary differential equations—Equations (11) and (12)—using the fourth-order Runge–Kutta method along the module’s flow channel. This numerical approach allows for quantifying the enhanced permeate flux resulting from increased turbulence intensity due to the insertion of eddy promoters. The bulk temperature distributions of the hot saline and cold feed streams, along with the membrane surface temperatures, were computed using a one-dimensional theoretical model. These results are presented in Figure 10, plotted along the axial coordinate, with different array configurations considered as parameters. Theoretical predictions reveal a gradual attenuation of both membrane surface and bulk fluid temperatures along the flow direction, leading to a reduction in the temperature driving force for vapor transport. However, the temperature gradients across the membrane surfaces are consistently larger in promoter-filled modules compared to those in empty-channel configurations. As a result, higher vapor flux is achieved in the promoter-filled modules due to the stronger driving force.

**Figure 11:**
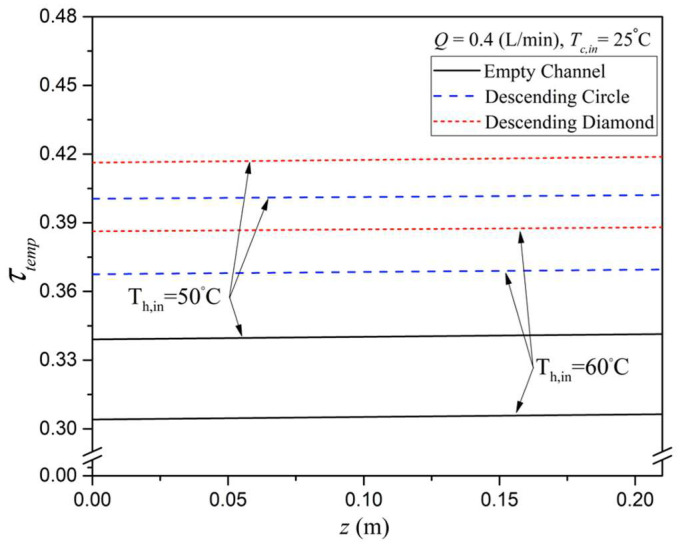
Effects of inlet hot saline feed temperature and array configurations on $\tau_{temp}.$.

In addition, theoretical predictions of the temperature polarization coefficient, $\tau_{temp\;}$, were obtained from Equation (10) and are illustrated in Figure 11, with the inlet hot saline temperature used as a parameter for both descending circle-shaped and diamond-shaped turbulence promoters. A noticeably higher $\tau_{temp\;}$ was predicted for modules with inserted eddy promoters, attributed to the suppression of temperature polarization effects through disruption of the thermal boundary layer on the membrane surface. As the inlet hot saline temperature increases, the associated rise in vapor pressure leads to higher permeate flux through the membrane. This is due to the reduced temperature difference across the membrane surfaces (i.e., $T_{1}^{p}-T_{2}^{p}$, as shown in Figure 6) and the corresponding decrease in $\tau_{temp}.$ The reduction in temperature polarization is more pronounced in diamond-shaped promoter-filled modules, resulting in a higher $\tau_{temp\;}$ value compared to the circle-shaped promoter modules, as demonstrated in Figure 11.

### 4.2. Permeate Flux Improvement by Inserting 3D-Printed Turbulence Promoters

The accuracy deviation of the theoretical predictions of permeate fluxes with respect to experimental results for all measurements of descending circle and diamond promoters as illustrations was calculated using the following definition [36]:

(21) $$E\;(\%)=\frac{1}{N_{exp}}\sum_{j=1}^{N_{exp}}\frac{\left|N_{theo,j}^{”}-N_{exp,j}^{”}\right|}{N_{exp,j}^{”}}\;$$

Moffat [36] determined the experimental uncertainty for each individual measurement from the experimental runs as follows:

(22) $$S_{N_{exp,j}^{”}}={\left\{\sum_{i=1}^{N_{exp}}\frac{{\left(N_{exp,j}^{”}-\bar{N_{exp,j}^{”}}\right)}^{2}}{N_{exp}-1}\right\}}^{1/2}\;$$

The mean value of the resulting uncertainty of the experimental measurements was defined by

(23) $$S_{\bar{N_{exp,j}^{”}}}=\frac{S_{N_{exp,j}^{”}}}{\sqrt{N_{exp}}}$$

where $N_{exp}$, $N_{theo,j}^{”}$, $N_{exp,j}^{”}$ and $\bar{N_{exp,j}^{”}}$ are the number of experimental runs, theoretical predictions, experimental results of permeate fluxes and mean value of experimental results of permeate fluxes, respectively. The deviation and uncertainty of the experimental measurements are well minimized within $3.1\times 10^{-3}\leq E\leq 8.31\times 10^{-2}$ and $4.94\times 10^{-3}\leq S_{\bar{J_{exp}}}\leq 7.58\times 10^{-3}$, respectively. It is seen from Table 2 that good agreement was expected between the theoretical predictions and experimental results.

**Figure 12:**
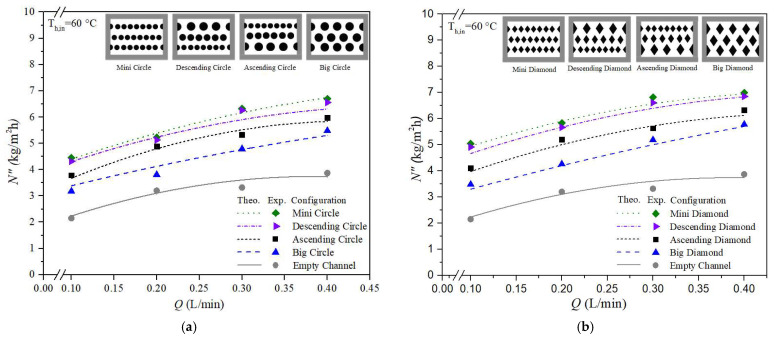
Comparisons of theoretical permeate fluxes under various promoter-filled channels. (**a**) Circle-type promoter-filled channels; (**b**) Diamond-type promoter-filled channels.

The increased temperature driving-force gradient across the membrane surfaces—denoted as $T_{1}^{p}$ and $T_{2}^{p}$, as shown in Figure 6b—results in higher permeate flux due to the enhanced turbulence intensity near the membrane interface. This enhancement, brought about by the insertion of eddy promoters into the hot saline feed stream, reduces the thermal boundary layer thickness and consequently leads to a larger temperature polarization coefficient, $\tau_{temp}$. This study graphically compares theoretical predictions of permeate flux for both circle-shaped and diamond-shaped promoter-filled channels with ascending and descending hydraulic diameter configurations. Improved device performance was observed in modules with ascending and descending promoter-filled channels, achieving higher permeate flux compared to modules using uniform promoter-filled channels, as illustrated in Figure 12a,b. The predictive approach used for estimating permeate flux can be extended to various geometric promoter types and array configurations. This was accomplished through the same regression procedure applied to determine the Nusselt number correlations for both empty-channel modules and promoter-filled channel modules. Experimental and theoretical results of permeate flux are shown in Figure 12a,b for diamond-type and circle-type turbulence promoters configured in ascending, descending, and uniform arrays. Permeate flux was found to increase with higher hot saline feed flow rates across all configurations. The agreement between theoretical predictions and experimental data is reasonably consistent, as demonstrated in Figure 12a,b. Furthermore, the temperature polarization effect is more pronounced in the latter half of the module compared to the first half. This indicates that descending promoter-filled channels yield higher permeate flux than ascending configurations due to better suppression of the boundary layer effects. Lastly, Figure 12a,b establish the rank order of permeate flux performance among the tested promoter configurations: mini promoters > descending promoters > ascending promoters > big promoters.

**Figure 13:**
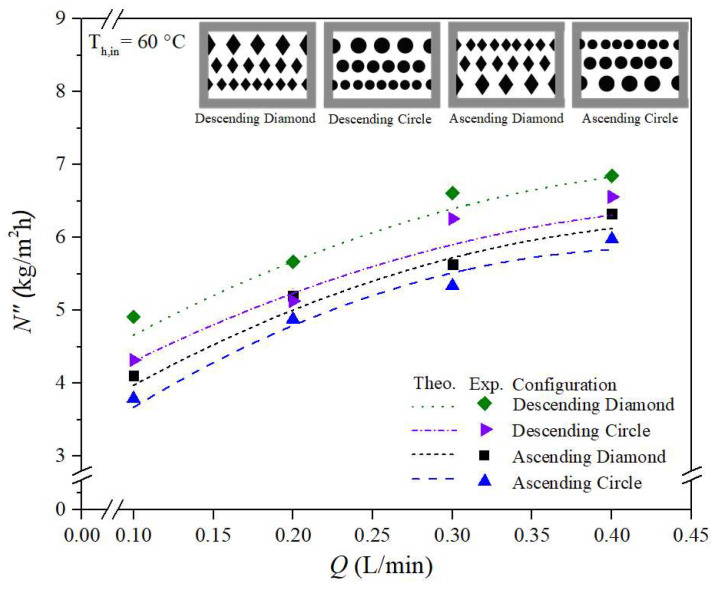
Effects of array configurations on permeate fluxes under both descending diamond- and circle-type turbulence promoters.

Moreover, the results also show that the module that uses diamond-type promoter-filled channels enhances more vortices and eddies significantly compared with modules that use circle-type promoter-filled channels. This is because of the non-smooth curvature shape of the obstacles of the diamond-type turbulence promoters, as depicted in Figure 13.

It is noteworthy that higher permeate flux was achieved at elevated inlet hot saline temperatures, with the order of flux magnitude as follows: 60 °C > 55 °C > 50 °C > 45 °C. This enhancement is attributed to the increased production of water vapor in the hot saline feed region, which results in a greater saturated vapor pressure gradient across the membrane. Additionally, a higher hot saline feed flow rate induces faster velocities and stronger vortices, which effectively reduce heat transfer resistance within the thermal boundary layer—particularly in modules equipped with descending promoter-filled channels under identical operating conditions. As expected, modules incorporating eddy promoters exhibited significant improvements in permeate flux compared to those utilizing no-promoter-filled (empty) channels.

The relative permeate flux improvement, $I_{N}$, was calculated as the percentage increase in comparison to the baseline module with an empty channel, as shown below:

(24) $$I_{N}\left(\%\right)=\frac{N_{promoter}^{”}-N_{empty}^{”}}{N_{empty}^{”}}\times 100,\;forpromote\mathrm{r-f}illedchannels$$

where the subscripts *promoter* and *empty* denote modules with and without inserted turbulence promoters, respectively. Theoretical predictions of permeate flux improvements, $I_{N}$, for modules with ascending, descending, and uniform hydraulic width configurations are summarized in Table 3, Table 4, Table 5 and Table 6, using inlet hot saline temperature and volumetric flow rate as variables. The analysis reveals that the incorporation of eddy promoters into the hot saline feed stream significantly enhances permeate flux compared to modules with empty channels due to the effective mitigation of the temperature polarization effect. Device performance using mini-promoter-filled channels was found to be superior to that using big-promoter-filled channels, as confirmed by the data in Table 3 and Table 4. Specifically, the relative increment in permeate flux reached up to 81.88% and 91.73% for descending circle-type and diamond-type turbulence promoters, respectively, when compared to the no-promoter-filled module. Furthermore, results from Table 3, Table 4, Table 5 and Table 6 indicate that permeate flux improvements with 3D-printed turbulence promoters increase with higher inlet saline temperatures but tend to decrease at higher feed flow rates. The insertion of promoters with both ascending and descending hydraulic diameters demonstrates a significant enhancement in permeate flux, confirming their technical viability for performance improvement in DCMD modules.

### 4.3. Heat Transfer Enhancement Factor

**Figure 14:**
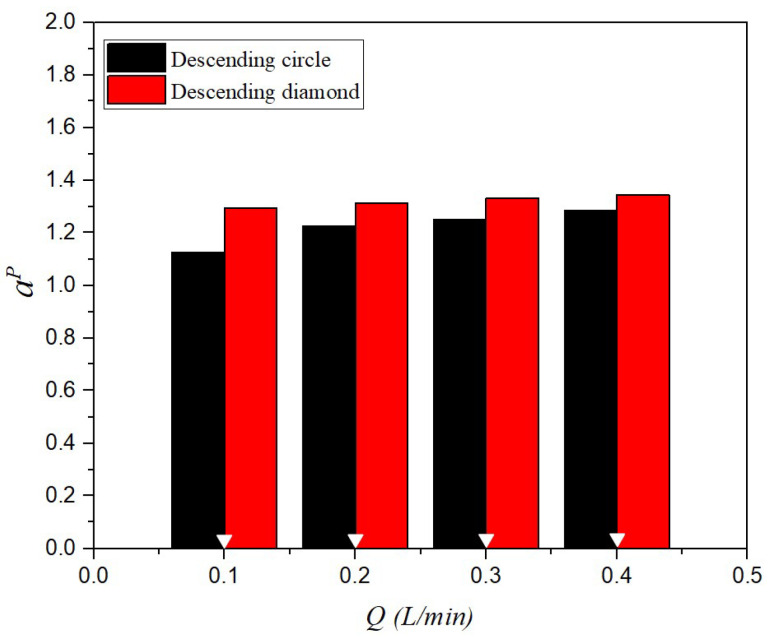
Comparisons of correlated Nusselt numbers for the channels with the insertion of both descending circle- and diamond-type turbulence promoters.

**Figure 15:**
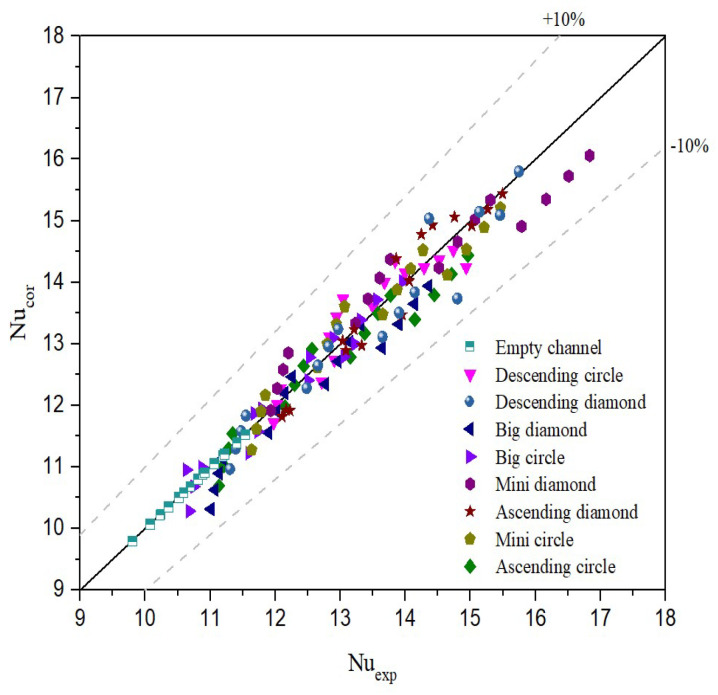
Deviations between the correlated and experimental Nusselt numbers.

The implementation of 3D-printed promoter-filled channels demonstrates strong technical feasibility and significantly enhances permeate flux. Results show that higher inlet saline feed temperatures lead to increased ${Nu}^{P}$ values, corresponding to higher heat transfer rates. The correlation expression for Nusselt numbers is applicable to both empty-channel modules and those with embedded eddy promoters. These promoters effectively disrupt the thermal boundary layer and intensify turbulence, resulting in improved heat transfer performance. A higher Nusselt number was achieved in promoter-filled modules compared to empty channels, reflecting an enhancement in the convective heat transfer coefficient. Consequently, the heat transfer enhancement factor, $\alpha^{P}$, is expressed using Equation (15), in conjunction with Equations (25) and (26), as illustrated in Figure 14 and the deviations between the correlated and experimental Nusselt numbers are within 10%, as shown in Figure 15.

(25) $${Nu}_{lam}=8.14{{\left(Re\right)}^{0.04}(Pr)}^{-0.44}$$

(26) $${Nu}^{P}=1.24{\left(\frac{W_{p}}{L}\right)}^{-0.03}{\left(R_{w}\right)}^{-0.16}{\left(Re\right)}^{0.17}{\left(Pr\right)}^{-0.01}$$

**Figure 16:**
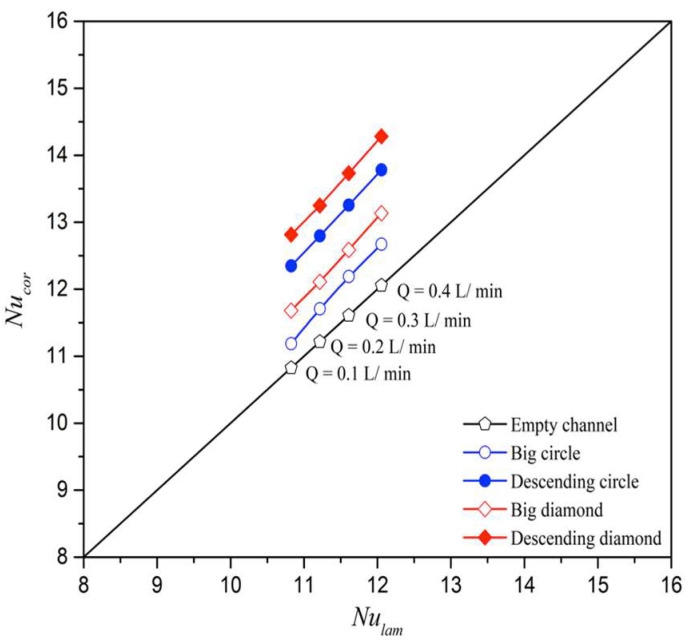
Comparisons between correlated and experimental Nusselt numbers for the no-promoter-filled and promoter-filled channels under various array configurations.

The heat transfer coefficients, expressed in terms of the correlated Nusselt numbers, were determined using a theoretical model and compared with a module without promoter-filled channels. The results align linearly with the experimental data, as shown in Figure 16.

**Figure 17:**
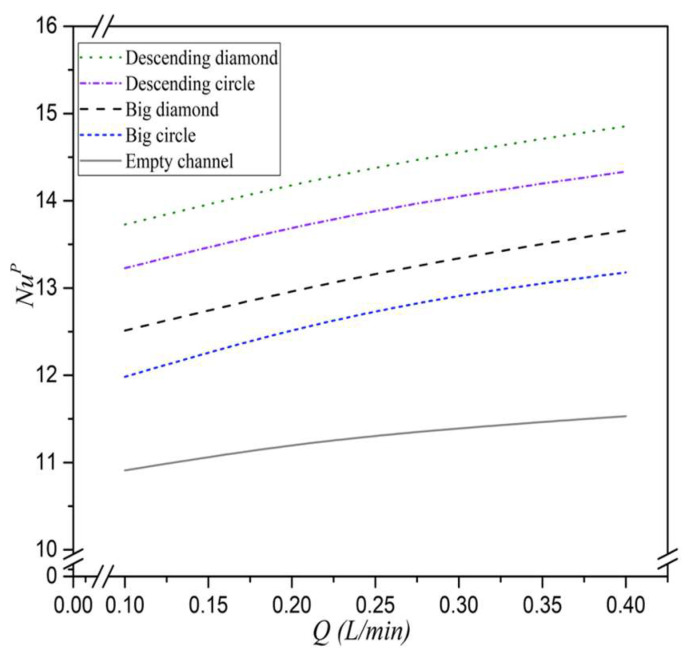
Effects of various array configurations and geometric shapes of 3D-printed turbulence promoters on Nusselt numbers.

Remarkably, the eddy promoters play a dominant role in disturbing the thermal boundary layer and reducing heat transfer resistance, which in turn boosts permeate flux. The correlated Nusselt numbers from Equation (26) show that modules with descending hydraulic diameters attain higher heat transfer rates than those using uniform promoter diameters or empty channels, as demonstrated in Figure 17. Additionally, modules incorporating diamond-type turbulence promoters generate more vortices and eddies compared to those with circle-type promoters. This is attributed to the non-smooth curvature of the diamond-shaped obstacles, which promote greater flow disruption.

Two geometric shapes of 3D-printed turbulence promoters—diamond-type and circle-type—were employed in membrane distillation modules, tested under eight different array configurations at various equivalent hydraulic diameters. Each promoter obstructed approximately 13% of the hydrophobic membrane surface area, which was considered unavailable for vapor flux transfer and was accounted for in the calculation procedure. The insertion of turbulence promoters effectively mitigated the temperature polarization effect by increasing turbulence intensity within the thermal boundary layer and enhancing shear stress on the membrane surface. Notably, modules utilizing diamond-type promoter-filled channels exhibited greater turbulence intensity—due to their non-smooth curvature—compared to circle-type configurations, thereby achieving higher permeate flux. The further permeate flux enhancement ($E_{P}$) was calculated based on the performance of descending promoter-filled modules relative to uniform promoter-filled modules operating under identical conditions. The enhancement was determined using the following relationship:

(27) $$E_{P}\left(\%\right)=\frac{N_{descending}^{”}-N_{uniform}^{”}}{N_{uniform}^{”}}\times 100\;=\left[\frac{\left(N_{descending}^{”}-N_{empty}^{”})-(N_{uniform}^{”}-N_{empty}^{”}\right)}{N_{empty}^{”}}\right]\left(\frac{N_{empty}^{”}}{N_{uniform}^{”}}\right)\times 100=\left(I_{N}^{des}-I_{N}^{uni}\right)/\left(1+I_{N}^{uni}\right)\times 100$$

where $N_{uniform}^{”}$ and $N_{descending}^{”}$ represent the permeate fluxes in the modules embedded with uniform and descending promoter-filled channels, respectively, for both circle-type and diamond-type promoters. Likewise, $I_{N}^{uni}$ and $I_{N}^{des}$ denote the permeate flux improvements in the corresponding modules. The percentage improvements in permeate flux ($I_{N}$) and further enhancements ($E_{p}$) for both circle-type and diamond-type turbulence promoters are summarized in Table 7**.** As illustrated, the implementation of descending promoter-filled channels in the hot saline feed stream led to noticeable enhancement in convective heat transfer coefficients, resulting in increased vapor transport across the membrane. The results indicate that further permeate flux enhancements increase with higher inlet saline temperatures but tend to decrease with increasing hot saline feed flow rates. A maximum further enhancement of **35.48%** was achieved using descending diamond-type promoter-filled channels, which outperformed their circle-type counterparts. This enhancement was calculated based on comparison with the uniform big-type promoter configuration.

Furthermore, the insertion provides the permeate flux improvement due to mitigating the thermal boundary layer with a smaller thermal resistance; therefore, Table 8 is presented to show the thermal resistance reduction under Reynolds numbers within 14.06 $\leq {Re}_{h}\leq 108.90$ under all array configurations. Moreover, the thermal resistance (or convection resistance) is defined as follows:

(28) $$R_{conv}=\frac{1}{h_{h}A}$$

The results show that the thermal resistances decrease with both the inlet hot saline temperatures and the hot saline feed flow rates, which is consistent with the decrement of transport resistances by inserting turbulence promoters.

### 4.4. Energy Consumption Increment

**Figure 18:**
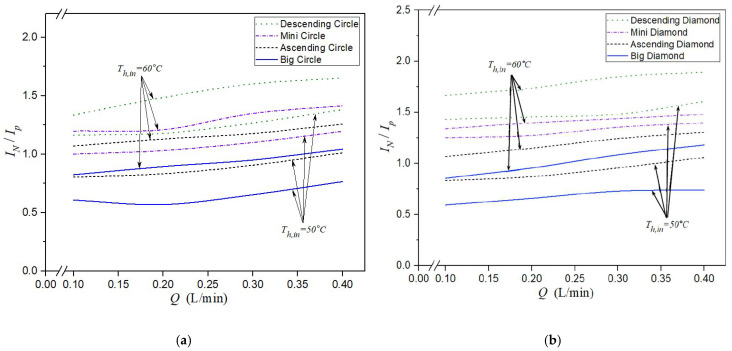
Comparisons of $I_{N}/I_{P}$ with the insertion of 3D-printed turbulence promoters under various array configurations. (**a**) Descending circle-type promoter-filled channels; (**b**) Descending diamond-type promoter-filled channels.

The ratio of permeate flux improvement to energy consumption increment, denoted as $I_{N}/I_{P},$ was used to evaluate the technical feasibility of promoter-filled channels from an economic perspective. This ratio helps identify optimal operating conditions for achieving enhanced permeate flux with reasonable energy costs. While the insertion of eddy promoters introduces additional frictional losses—depending on the array configuration—the trade-off can be justified by the corresponding increase in the permeate flux. The results of $I_{N}/I_{P}$ demonstrate that an increase in the percentage of the permeate flux improvement can effectively offset the rise in the percentage of the power consumption increment by simply increasing the hot saline flow rate and inlet temperature under various array configurations, as shown in Figure 18a,b. A higher $I_{N}/I_{P}$ ratio indicates that the relative percentage increment of the permeate flux improvement is sufficient to counterbalance the additional percentage increment of the hydraulic dissipated power. In addition, the larger the value of $I_{N}/I_{P}$ obtained, the higher the permeate flux improvement achieved. This is particularly evident in modules with descending promoter-filled channels, which outperform uniform promoter modules in terms of energy efficiency and overall device performance. As expected, modules embedded with diamond-type promoters exhibited better performance than those with circle-type promoters. Implementing uniform mini-promoter-filled channels achieved the highest permeate flux ($I_{N}$) due to their ability to induce stronger turbulence, as seen in Figure 12a,b. However, when considering the ratio $I_{N}/I_{P}$, descending promoter-filled channels outperformed uniform mini-promoter configurations, indicating better energy-to-performance efficiency. This reversal in ranking highlights the importance of evaluating both flux improvement and energy cost. In other words, both descending and ascending promoter-filled channels offer greater potential for efficient permeate flux improvement compared to that of using uniform promoter-filled channels, aligning better with economic and operational considerations.

## 5. Conclusions

Theoretical predictions of permeate flux were validated through experimental results under varying hot saline feed flow rates, inlet temperatures, geometric shapes of turbulence promoters, and array configurations. An assessment of the membrane effectiveness of implementing turbulence promoters was conducted to determine the permeate flux improvement that balances the desirable increment of the heat transfer coefficient and the undesirable decrement of the mass transfer surface area. Overall, the insertion of 3D-printed turbulence promoters into the flow channel exhibits significant potential for substantially augmenting permeate flux in the DCMD module due to mitigating the thermal boundary layer with a smaller thermal resistance and temperature polarization effect as well. The advantages of promoter-filled channels were comprehensively demonstrated, particularly with ascending and descending hydraulic diameter configurations in DCMD modules. The key conclusions drawn from this study are as follows:

1. Implementing promoter-filled channels with descending hydraulic diameters in the hot saline feed stream resulted in a significant relative permeate flux improvement. A maximum enhancement of 91.73% was achieved at an inlet temperature of 60 °C and a flow rate of 0.4 L/min.
2. Modules using uniform mini-type promoter-filled channels exhibited greater permeate flux improvements ($I_{N}$) compared to those using both ascending and descending promoter configurations. However, the ratio of $I_{N}/I_{P}$, which normalizes performance by hydraulic effect, showed an inverse trend among different promoter types.
3. Permeate flux improvements were more pronounced in modules with descending promoter-filled channels compared to ascending ones due to the development of a stronger and more uniform driving-force temperature gradient along the membrane module.
4. Among various array configurations, descending promoter-filled channels showed a clearly positive effect on permeate flux. This is attributed to enhanced temperature gradients achieved by strategically varying the hydraulic diameters, thereby optimizing thermal boundary layer disruption.

The regressed Nusselt number correlations derived from the theoretical model offer practical insights for designing and operating more efficient DCMD modules. The integration of 3D-printed turbulence promoters effectively enhances hydrodynamic conditions and heat transfer, validating both the technical feasibility and performance benefits of this approach. This work emphasizes the potential of exploring various geometric shapes and array configurations of eddy promoters for optimizing DCMD operations. The methodology presented can also be extended to a range of membrane separation processes, offering a foundation for future investigations with consideration of both technical and economic factors.

## Figures and Tables

**Table 1:** The average widths of various array configurations of circle- and diamond-type promoters.

Array Configurations	$N_{P}$	$W_{P}$ (mm)	$R_{w}$
Big circle	4	9.76	1.57
Medium circle	6	6.51	1.28
Mini circle	8	4.88	1.11
Ascending circle	8	4.88	1.43
Descending circle	4	9.76	1.43
Big diamond	4	9.77	1.80
Medium diamond	6	6.52	1.47
Mini diamond	8	4.89	1.28
Ascending diamond	8	4.89	1.64
Descending diamond	4	9.77	1.64

**Table 2:** The accuracy deviation between theoretical predictions $N_{theo}^{”}\;$ and experimental results $N_{exp}^{”}\;$ of absorption fluxes.

$T_{h,in}$ (°C **)**	Q **(L** **/** **min)**	Promoter-Filled Channel Configurations with Descending Hydraulic Diameter					
		Circle-Type			Diamond-Type		
		$N_{exp}^{”}$ (kg m^−2^ h^−1^)	$N_{theo}^{”}$ (kg m^−2^ h^−1^)	E (%)	$N_{exp}^{”}$ (kg m^−2^ h^−1^)	$N_{theo}^{”}$ (kg m^−2^ h^−1^)	E (%)
45	0.1	1.98	2.08	5.43	2.21	2.09	5.05
	0.2	2.49	2.47	3.72	2.42	2.51	0.80
	0.3	2.75	2.67	6.78	2.95	2.75	2.91
	0.4	3.21	3.02	8.31	3.37	3.09	5.92
50	0.1	2.74	2.82	3.09	2.91	2.82	2.92
	0.2	3.52	3.45	3.51	3.42	3.54	1.99
	0.3	3.87	3.97	1.21	4.12	4.07	2.58
	0.4	4.13	4.23	3.34	4.49	4.34	2.42
55	0.1	3.27	3.38	1.17	3.42	3.46	3.36
	0.2	4.21	4.19	2.27	4.41	4.31	0.48
	0.3	5.05	4.86	3.62	5.25	5.06	3.76
	0.4	5.74	5.41	8.01	6.12	5.63	5.75
60	0.1	4.32	4.62	0.81	4.91	4.87	6.94
	0.2	5.13	5.28	4.45	5.84	5.58	2.92
	0.3	6.26	6.05	0.31	6.54	6.56	3.35
	0.4	6.56	6.28	5.88	6.46	6.84	4.27

**Table 3:** Effects of promoter-filled operations on permeate flux improvements $I_{N}$.

$T_{h,in}$ (°C **)**	Q **(L** **/** **min)**	Promoter-Filled Channel Configurations with Uniform Big-Type Promoters				
		Empty Channel	Circle-Type		Diamond-Type	
		$N_{empty}^{”}$ (kg m^−2^ h^−1^)	$N_{circle}^{”}$ (kg m^−2^ h^−1^)	$I_{N}$ (%)	$N_{diamond}^{”}$ (kg m^−2^ h^−1^)	$I_{N}$ (%)
45	0.1	1.29	1.59	23.25	1.70	31.78
	0.2	1.58	1.93	22.15	2.05	29.74
	0.3	1.76	2.11	19.88	2.26	28.40
	0.4	2.04	2.40	17.64	2.57	25.98
50	0.1	1.63	2.11	29.44	2.24	37.42
	0.2	2.11	2.67	26.54	2.82	33.64
	0.3	2.47	3.11	25.91	3.26	31.98
	0.4	2.67	3.33	24.71	3.49	30.71
55	0.1	1.88	2.51	33.51	2.74	45.74
	0.2	2.43	3.22	32.51	3.42	40.74
	0.3	2.91	3.78	29.89	4.02	38.14
	0.4	3.32	4.23	27.40	4.48	34.93
60	0.1	2.54	3.41	34.25	3.78	48.81
	0.2	2.94	3.94	34.01	4.34	47.61
	0.3	3.49	4.65	33.23	5.12	46.70
	0.4	3.67	4.88	32.97	5.34	45.50

**Table 4:** Effects of promoter-filled operations on permeate flux improvements $I_{N}$.

$T_{h,in}$ (°C **)**	Q **(L** **/** **min)**	Promoter-Filled Channel Configurations with Uniform Mini-Type Promoters				
		Empty Channel	Circle-Type		Diamond-Type	
		$N_{empty}^{”}$ (kg m^−2^ h^−1^)	$N_{circle}^{”}$ (kg m^−2^ h^−1^)	$I_{N}$ (%)	$N_{diamond}^{”}$ (kg m^−2^ h^−1^)	$I_{N}$ (%)
45	0.1	1.29	2.23	72.86	2.44	89.14
	0.2	1.58	2.57	62.66	2.88	82.27
	0.3	1.76	2.81	59.65	3.19	81.25
	0.4	2.04	3.24	58.82	3.68	80.39
50	0.1	1.63	2.89	77.30	3.16	93.86
	0.2	2.11	3.52	66.82	3.94	86.72
	0.3	2.47	4.02	62.75	4.57	85.02
	0.4	2.67	4.23	58.42	4.91	83.89
55	0.1	1.88	3.37	79.25	3.62	92.55
	0.2	2.43	4.21	73.25	4.66	91.76
	0.3	2.91	4.86	67.01	5.53	90.03
	0.4	3.32	5.39	62.34	6.23	87.65
60	0.1	2.54	4.63	82.28	4.92	93.70
	0.2	2.94	5.31	80.61	5.67	92.85
	0.3	3.49	6.27	79.65	6.59	88.82
	0.4	3.67	6.52	77.65	6.89	87.73

**Table 5:** Effects of promoter-filled operations on permeate flux improvements $I_{N}$.

$T_{h,in}$ (°C **)**	Q **(L** **/** **min)**	Promoter-Filled Channel Configurations with Descending Hydraulic Diameters				
		Empty Channel	Circle-Type		Diamond-Type	
		$N_{empty}^{”}$ (kg m^−2^ h^−1^)	$N_{circle}^{”}$ (kg m^−2^ h^−1^)	$I_{N}$ (%)	$N_{diamond}^{”}$ (kg m^−2^ h^−1^)	$I_{N}$ (%)
45	0.1	1.29	2.08	61.24	2.09	62.02
	0.2	1.58	2.47	56.32	2.51	58.86
	0.3	1.76	2.67	51.70	2.75	56.25
	0.4	2.04	3.02	48.03	3.09	51.47
50	0.1	1.63	2.82	73.01	2.82	73.01
	0.2	2.11	3.45	63.50	3.54	67.77
	0.3	2.47	3.97	60.72	4.07	64.77
	0.4	2.67	4.23	58.42	4.34	62.54
55	0.1	1.88	3.38	79.78	3.46	84.04
	0.2	2.43	4.19	72.42	4.31	77.36
	0.3	2.91	4.86	67.01	5.06	73.88
	0.4	3.32	5.41	62.95	5.63	69.57
60	0.1	2.54	4.62	81.88	4.87	91.73
	0.2	2.94	5.28	79.59	5.58	89.79
	0.3	3.49	6.05	73.35	6.56	87.96
	0.4	3.67	6.28	71.11	6.84	86.37

**Table 6:** Effects of promoter-filled operations on permeate flux improvements $I_{N}$.

$T_{h,in}$ (°C **)**	Q **(L** **/** **min)**	Promoter-Filled Channel Configurations with Ascending Hydraulic Diameters				
		Empty Channel	Circle-Type		Diamond-Type	
		$N_{empty}^{”}$ (kg m^−2^ h^−1^)	$N_{circle}^{”}$ (kg m^−2^ h^−1^)	$I_{N}$ (%)	$N_{diamond}^{”}$ (kg m^−2^ h^−1^)	$I_{N}$ (%)
45	0.1	1.29	1.78	37.98	1.84	42.63
	0.2	1.58	2.16	36.71	2.23	41.13
	0.3	1.76	2.35	33.52	2.46	39.77
	0.4	2.04	2.71	32.84	2.83	38.72
50	0.1	1.63	2.32	42.33	2.35	44.17
	0.2	2.11	2.99	41.71	3.03	43.60
	0.3	2.47	3.41	38.05	3.51	42.10
	0.4	2.67	3.64	36.32	3.74	40.07
55	0.1	1.88	2.73	45.21	2.94	56.38
	0.2	2.43	3.49	43.62	3.76	54.73
	0.3	2.91	4.15	42.61	4.41	51.54
	0.4	3.32	4.67	40.66	4.85	46.08
60	0.1	2.54	3.85	51.57	4.05	59.44
	0.2	2.94	4.43	50.68	4.65	58.16
	0.3	3.49	5.15	47.56	5.51	57.87
	0.4	3.67	5.34	45.50	5.74	56.40

**Table 7:** Theoretical predictions of permeate flux improvements and further permeate flux enhancement in the module with descending promoter-filled channels.

$T_{h,in}$ **(** °C **)**	Q **(L/** **min)**	Descending Promoter-Filled Channels					
		Big-Circle	Big-Diamond	Descending Circle-Type		Descending Diamond-Type	
		$I_{N}$ (%)	$I_{N}$ (%)	$I_{N}$ (%)	$E_{P}(\%)$	$I_{N}$ (%)	$E_{P}$ (%)
45	0.1	23.25	31.78	61.24	22.94	62.02	30.81
	0.2	22.15	29.74	56.32	22.43	58.86	27.97
	0.3	19.88	28.40	51.70	21.68	56.25	26.54
	0.4	17.64	25.98	48.03	20.23	51.47	25.83
50	0.1	29.44	37.42	73.01	25.89	73.01	33.64
	0.2	26.54	33.64	63.50	25.53	67.77	29.21
	0.3	25.91	31.98	60.72	24.84	64.77	27.65
	0.4	24.71	30.71	58.42	24.35	62.54	27.02
55	0.1	33.51	45.74	79.78	26.27	84.04	34.66
	0.2	32.51	40.74	72.42	26.023	77.36	30.12
	0.3	29.89	38.14	67.01	25.87	73.88	28.57
	0.4	27.40	34.93	62.95	25.66	69.57	27.89
60	0.1	34.25	48.81	81.88	28.83	91.73	35.48
	0.2	34.01	47.61	79.59	28.57	89.79	34.01
	0.3	33.23	46.70	73.35	28.12	87.96	30.10
	0.4	32.97	45.50	71.11	28.08	86.37	28.68

**Table 8:** Thermal resistances of modules with/without inserting turbulence promoters.

$T_{h,in}$(°C)	Q(L/min)	Thermal Resistances (or Convection Resistance) (K/W)						
		Circle-Type				Diamond-Type		
		Empty Channel	Big	Descending	Mini	Big	Descending	Mini
45	0.1	3.64	2.90	2.65	2.48	2.76	2.36	2.35
	0.2	3.56	2.81	2.58	2.41	2.68	2.30	2.28
	0.3	3.49	2.73	2.51	2.35	2.60	2.24	2.22
	0.4	3.42	2.65	2.45	2.30	2.53	2.19	2.17
50	0.1	3.53	2.75	2.55	2.38	2.62	2.27	2.25
	0.2	3.45	2.67	2.48	2.33	2.54	2.21	2.19
	0.3	3.38	2.60	2.41	2.27	2.47	2.16	2.14
	0.4	3.31	2.53	2.35	2.22	2.41	2.11	2.09
55	0.1	3.42	2.58	2.45	2.30	2.46	2.19	2.17
	0.2	3.35	2.51	2.38	2.24	2.39	2.13	2.11
	0.3	3.28	2.45	2.33	2.19	2.33	2.09	2.06
	0.4	3.21	2.38	2.27	2.14	2.27	2.04	2.01
60	0.1	3.31	2.49	2.35	2.22	2.38	2.11	2.09
	0.2	3.25	2.45	2.30	2.16	2.33	2.06	2.04
	0.3	3.18	2.40	2.24	2.12	2.28	2.01	1.99
	0.4	3.12	2.35	2.19	2.07	2.24	1.97	1.94

## Data Availability

The original contributions presented in this study are included in the article. Further inquiries can be directed to the corresponding author.

## Abbreviations

$a_{w}$	Water activity in NaCl solution
A	Available membrane surface area (m^2^)
C	Friction losses coefficient
$C_{P}$	Heat capacity ($J\;kg^{-1}\;K^{-1}$)
$c_{k}$	Membrane coefficient based on the Knudsen diffusion model ($\mathrm{mol}\;m^{-2}Pa^{-1}s^{-1}$)
$c_{M}$	Membrane coefficient based on the molecular diffusion model ($\mathrm{mol}\;m^{-2}Pa^{-1}s^{-1}$)
$c_{m}$	Membrane permeation coefficient ($\mathrm{mol}\;m^{-2}Pa^{-1}s^{-1}$)
$D_{p}$	Turbulence promoter thickness (m)
$D_{m}$	Diffusion coefficient of air and vapor in the membrane ($m^{2}s^{-1}$)
${De}_{h,i}$	Hydraulic equivalent diameter of channel (m), i=h,c
E	Accuracy deviation of experimental results from the theoretical predictions
$f_{F,i}$	Fanning friction factor, i=h,c
$h_{c}$	Heat transfer coefficient of cold feed (W $m^{-2}K^{-1}$)
$h_{h}$	Heat transfer coefficient of hot saline feed (W $m^{-2}K^{-1}$)
$h_{h}^{p}$	Improved heat transfer coefficient of hot stream with promoter insertion (W $m^{-2}K^{-1}$)
$H_{i}$	Hydraulic dissipate energy (W), i=h,c
$I_{N}$	Permeate flux relative factor
$I_{p}$	Power consumption relative index
k	Thermal conductivity of water ($Wm^{-1}K^{-1}$)
L	Channel length (m)
$lw_{f,j}$	Friction loss (J kg^−1^), i=h,c
$M_{W}$	Molecular weight of water (kg mol^−1^)
$M_{W-air}$	Average molecular weight of water and air (kg mol^−1^)
$N_{p}$	The number of the flow segments under various configurations
$N^{\prime \prime }$	Permeate flux ($\mathrm{kg}\;m^{-2}s$)
$\bar{N^{\prime \prime }}$	Average value of $N^{\prime \prime }$ for all numbers of experimental measurements ($\mathrm{kg}\;m^{-2}s$)
$N_{exp}$	Number of experimental measurements
${Nu}^{p}$	Enhanced dimensionless Nusselt number
${Nu}_{lam}$	Nusselt number for laminar flow
$P_{1}$	Vapor pressure at membrane surface in the hot saline feed (Pa)
$P_{2}$	Vapor pressure at membrane surface in the cold feed (Pa)
$P_{T}$	Pressure at the temperature *T* (Pa)
$P_{m}$	Mean saturated pressure in membrane (Pa)
$P^{sat}$	Saturation vapor pressure (Pa)
Q	Volumetric flow rate (m^3^ s^−1^)
$q^{\prime \prime }$	Heat flux ($J\;m^{-2}s^{-1}$)
$r_{p}$	Membrane pore radius (m)
R	Gas constant (8.314 J mol^−1^ K^−1^)
$R_{\mathrm{conv}}$	Thermal resistance (K W^−1^)
Re⁡	Reynolds number
$R_{w}$	The ratio of the largest width of each promoter to the average big-circle promoter width
$S_{N^{\prime \prime }}$	Uncertainty of the experimental measurements
$S_{\bar{N^{\prime \prime }}}$	Mean value of $S_{N^{\prime \prime }}$
$T_{1}$	Membrane surface temperature in the hot saline feed region (K)
$T_{2}$	Membrane surface temperature in the cold feed region (K)
$T_{1}^{p}$	Membrane surface temperature with promoter insertion in the hot saline feed region (K)
$T_{2}^{p}$	Membrane surface temperature with promoter insertion in the cold feed region (K)
$T_{m}$	Average membrane temperature (K)
$\bar{v}$	Average velocity ($m\;s^{-1}$)
W	Width of channel (m)
$W_{p}$	Pseudo-average width occupied by turbulence promoters in each flow segment (m)
$x_{w}$	Liquid mole fraction of water
$x_{\mathrm{NaCl}}$	Mole fraction of NaCl in saline solution
$Y_{w}$	Vapor mole fraction of water
${\left|Y_{m}\right|}_{ln}$	Natural log mean vapor mole fraction of water in the membrane
z	Axial coordinate along the flow direction (m)
Greek letters	
$\alpha^{p}$	Enhancement factor
$\delta_{m}$	Thickness of membrane (µm)
ε	Membrane porosity
$\eta_{v}$	Gas viscosity ($\mathrm{Ns}\;m^{-2}$)
λ	Latent heat of water ($J\;kg^{-1}$)
μ	Viscosity ($\mathrm{Ns}\;m^{-2}$)
ρ	Density ($\mathrm{kg}\;m^{-3}$)
$\sigma_{w}$	Diffusion volume of water ($m^{3}$)
$\sigma_{air}$	Diffusion volume of water ($m^{3}$)
τ	Membrane tortuosity
$\tau_{temp\;}$	Temperature polarization coefficients
Subscripts	
1	Membrane surface on hot fluid side
2	Membrane surface on cold fluid side
*c*	Cold feed stream
*h*	Hot feed stream
*cor*	Correlated results
*empty*	Channel without embedded turbulence promoters
*exp*	Experimental results
*in*	At the inlet
lam	Laminar flow
*m*	Membrane
*out*	At the outlet
*promoter*	Channel with embedded turbulence promoters
*theo*	Theoretical predictions
Superscripts	
*h*	Heat transfer mechanism
*p*	Promoter-filled channels
